# *Yersinia pestis* and plague: an updated view on evolution, virulence determinants, immune subversion, vaccination, and diagnostics

**DOI:** 10.1038/s41435-019-0065-0

**Published:** 2019-04-03

**Authors:** Christian E. Demeure, Olivier Dussurget, Guillem Mas Fiol, Anne-Sophie Le Guern, Cyril Savin, Javier Pizarro-Cerdá

**Affiliations:** 1 Yersinia Research Unit, Institut Pasteur, F-75724 Paris, France; 2 Université Paris-Diderot, Sorbonne Paris Cité, F-75013 Paris, France; 3 National Reference Laboratory ‘Plague & Other Yersiniosis’, Institut Pasteur, F-75724 Paris, France; 4 World Health Organization Collaborating Research & Reference Centre for Yersinia, Institut Pasteur, F-75724 Paris, France

**Keywords:** Bacterial infection, Infection

## Abstract

Plague is a vector-borne disease caused by *Yersinia pestis*. Transmitted by fleas from rodent reservoirs, *Y. pestis* emerged <6000 years ago from an enteric bacterial ancestor through events of gene gain and genome reduction. It is a highly remarkable model for the understanding of pathogenic bacteria evolution, and a major concern for public health as highlighted by recent human outbreaks. A complex set of virulence determinants, including the *Yersinia* outer-membrane proteins (Yops), the broad-range protease Pla, pathogen-associated molecular patterns (PAMPs), and iron capture systems play critical roles in the molecular strategies that *Y. pestis* employs to subvert the human immune system, allowing unrestricted bacterial replication in lymph nodes (bubonic plague) and in lungs (pneumonic plague). Some of these immunogenic proteins as well as the capsular antigen F1 are exploited for diagnostic purposes, which are critical in the context of the rapid onset of death in the absence of antibiotic treatment (less than a week for bubonic plague and <48 h for pneumonic plague). Here, we review recent research advances on *Y. pestis* evolution, virulence factor function, bacterial strategies to subvert mammalian innate immune responses, vaccination, and problems associated with pneumonic plague diagnosis.

## Introduction

Plague is a vector-borne illness transmitted by fleas to a variety of wildlife rodents, which represent natural reservoirs for the disease in a wide range of habitats around the world [1]. The etiological agent of plague is the Gram-negative bacterium *Yersinia pestis* [2], discovered by the Institut Pasteur, bacteriologist Alexandre Yersin during a plague outbreak in Hong Kong in 1894 [3]. Plague has impacted the history of humankind through several pandemics that have initially spread from Central Asia to Africa and Europe, and plague has reached every continent during the last 150 years [4]. In the 21st century, plague is present in Asia, Africa and America [5], and recent outbreaks in Uganda [6], China [7], Democratic Republic of Congo [8], and Madagascar [9, 10] remind that plague is still a major public health concern.

*Y. pestis* is highly similar on a genomic level to the enteric pathogen *Y. pseudotuberculosis*; however, a series of gene gain and gene loss events have led to the appearance of markedly different mechanisms of disease as well as niche preference and lifestyle [11]. *Y. pestis* displays a quite unique set of virulence factors that allow successful infection of fleas and subversion of immune responses in mammalian hosts, leading to rapid host death in the absence of adequate treatment. In this article, we review recent advances in plague research, particularly in the fields of evolution, virulence determinants, and subversion of mammalian immune responses, as well as an overview of the critical aspects of vaccination and pneumonic plague diagnosis. For other aspects of *Y. pestis* infection, such as *Y. pestis* adaptation to the flea, see, e.g., the review by Hinnebusch et al. [12].

## Emergence and evolution of *Yersinia pestis*

Insights into *Y. pestis* phylogeny are emerging from ancient DNA (aDNA) paleo-genomic studies, which have been positively impacted by the development of high-throughput, “next-generation” DNA sequencing (NGS) technologies, together with an impressive decline in sequencing costs. The recovery of *Y. pestis* DNA from the teeth of prehistoric individuals has uncovered the branching of independent lineages throughout Eurasia in the Neolithic, and allowed to accurately estimate that *Y. pestis* diverged from *Y. pseudotuberculosis* around 5700–6000 years ago (ya) [13–16]. This emergence was characterized by the acquisition of the two virulence-associated plasmids pFra/pMT1 and pPla/pPCP1 and by the inactivation (promoter mutation) of the virulence-associated gene *pde3* [15, 17].

**Fig. 1:**
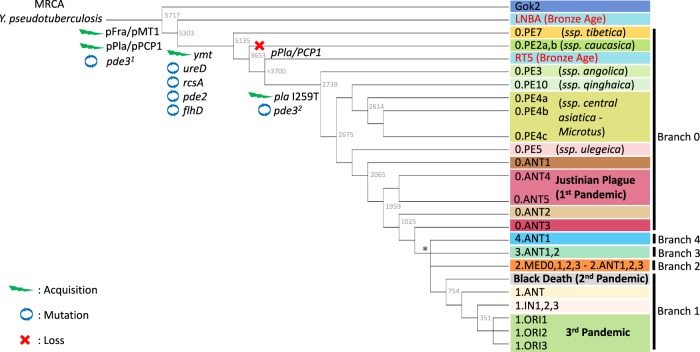
*Yersinia pestis* phylogenetic tree. Schematic representation of the *Y. pestis* genealogy, including previously described phylogenetic branches (0–4) and biovars (ANT: Antiqua, MED: Medievalis, ORI: Orientalis, IN: Intermediate, PE: Pestoides, including Microtus isolates), as well as six novel Pestoides clusters [25]. The length of the branches does not reflect the evolutionary time. Nodes from branch 3, branch 2 and branch 1.IN were collapsed for simplicity. The node indicated as an asterisk marks the polytomy known as the «Big Bang», which gave rise to branches 1–4. The three historically recorded plague pandemics are presented in bold next to their associated phylogenetic lineages. The key gene gain and inactivation events in the evolution of *Y. pestis* from *Y. pseudotuberculosis* are displayed. These events include the acquisition of two virulence-associated plasmids (pFra/pMT1 and pPla/pPCP1), the gain of *Yersinia* murine toxin (*ymt*) in the pFra/pMT1 plasmid, as well as by the inactivation of the virulence-associated genes *pde3* (^1^promoter mutation; ^2^inactivating mutation), *ureD*, *rcsA*, *flhD,* and *pde2*. The aftermath of these molecular changes gave rise to the *Y. pestis*’ flea-borne transmission and thereby the ability to cause bubonic plague. The acquisition of the I259T mutation in the plasminogen activator (Pla), a key virulence factor responsible for the fulminant lung infection specific of *Y. pestis*, is also presented in the phylogeny

Whole-genome-based phylogenies have defined a five-branch population structure for *Y. pestis* [18], following the previously described nomenclatures that termed the lineages according to their phylogenetic branch (0–4) plus the biovar abbreviation: ANT (biovar Antiqua), MED (Medievalis), ORI (Orientalis), IN (Intermediate), and PE (Pestoides, including Microtus isolates). An early divergence event occurred between 5000 and 5700 ya, giving origin to the most basal *Y. pestis* lineages persisting today (0.PE7 and 0.PE2), and to two extinct Neolithic and Bronze Age lineages (Gok2 and LNBA, respectively) [14]. These latter ones appeared to have functional variants of the virulence-associated genes *ureD*, *rcsA*, *flhD*, and *pde2* as well as to lack the *Yersinia* murine toxin encoded by the *ymt* gene (Fig. 1). The acquisition of *ymt* [17] and inactivation of *ureD*, *rcsA*, *flhD, pde2*, and *pde3* critically contributed to the transmissibility of *Y. pestis* by fleas [19]. Interestingly, the plasminogen activator Pla, a key virulence factor responsible for the fulminant lung infection specific of *Y. pestis*, was carried by Gok2, LNBA, 0.PE2, and 0.PE7 lineages in its ancestral form (I259), which has been associated with reduced bacterial dissemination in mammals [20]. This suggests that these strains may have had a limited flea-borne transmissibility and a reduced invasiveness within the host [13, 15].

A second expansion event around 4000 ya gave rise to an extinct Bronze Age lineage (RT5) and to lineages persisting to the present day: 0.PE4 (*microtus*) and the diverse lineages that led to all known plague pandemics [16]. The ancestor in this polytomy incorporated *ymt* and had the derived, inactive forms of *ureD*, *rcsA*, *flhD*, and *pde2*, suggesting that the flea-mediated transmission mechanism causing bubonic plague evolved as early as 5000 ya [16]. The frequent presence of *Y. pestis* among Neolithic human remains indicates that this pathogen was prevalent and virulent, hypothesizing the existence of prehistoric plague pandemics [14]. Several models for early plague dispersion have been proposed [13, 14, 16], although the exact birthplace of the successful *Y. pestis* lineage that led to the historically recorded pandemics remains unclear.

Paleo-genomic studies also provided unambiguous evidence confirming the presence of *Y. pestis* in two historically recorded plague pandemics: the Justinian’s Plague (6–8th centuries) [21, 22] and the second plague pandemic (14–18th centuries) including the infamous Black Death [23, 24]. The phylogenetic reconstruction of Justinian’s Plague aDNA revealed novel, extinct Justinian lineages (0.ANT4 and 0.ANT5) that branched between current 0.ANT1 and 0.ANT2 lineages, inaugurating independent lineages from strains associated with subsequent pandemics [22]. The wide distribution of 0.ANT strains (0.ANT3 and the newly found 0.ANT5 population) within the high-mountain foci from Kyrgyzstan suggests a Central Asian origin for the Justinian’s Plague pandemic [25]. In contrast to the first plague pandemic genomes, all recovered Black Death strains cluster at the root of branch 1, near a *big bang* polytomy that gave rise to most of the extant strain diversity [18, 26], including the lineages responsible for the Modern plague pandemic [22].

Several scenarios have been proposed to explain the transmission dynamics and persistence of plague in Europe after its introduction from Central/Eastern Asia during the Black Death. Initial paleo-genomic analyses using medieval *Y. pestis* genomes suggested that different bacterial clones were responsible for the second pandemic [24], proposing that plague was repeatedly introduced in Western Europe from reservoirs located in Eastern Europe/Central Asia [27] and spread via commercial trade routes and human migrations [28]. Other studies have found limited *Y. pestis* diversity during the second pandemic and propose a different scenario, in which plague may have established in natural rodent-based foci within Europe that evolved and persisted until the 18th century, spreading eastward back to Central and East Asia to become the source of the contemporary epidemics [29, 30]. A recent study combining historical, archeological, and genomic data has reevaluated all previously published Medieval strains together with five novel *Y. pestis* genomes, and infers different waves of introduction of plague to Western Europe during medieval times [26]. Both long-term persistence together with multiple introductions might have shaped the dynamics of plague during the second pandemic [31], which could have also been transmitted by vectors that were different from those of rodents, such as human ectoparasites [32].

The third pandemic originated around 1855 in the Chinese province of Yunnan with the emergence of the biovar Orientalis strains, which belong to the 1.ORI branch. From this origin, plague spread worldwide via steamships carrying infected rats and is nowadays present in Asia, Africa, and America [4]. The transmission dynamics in the third pandemic were characterized by single introduction events, followed by a rapid expansion and settlement in local rodent reservoirs, which allowed a rodent–flea–rodent transmission cycle. This combination seems essential for plague establishment in newly colonized countries. For instance, this scenario took place with the introduction of plague in Brazil through the seaport of Santos, Sao Paulo in 1899 or in Toamasina, the main seaport of Madagascar, in 1898. Local settlement and expansion explain the observed diversity in the different 1.ORI lineages (1.ORI1, 1.ORI2, and 1.ORI3), each one representing a large wave of *Y. pestis* spread [4, 33–35]. The characterization of strains from extant natural plague foci has also contributed to sketch the historical transmission and the genealogy of *Y. pestis*. In light of the recent genotyping of 359 modern *Y. pestis* strains from the natural plague foci in the former Soviet Union countries, an update of the intraspecific taxonomy was proposed [25] (Fig. 1). This new population structure defined seven subspecies: the ssp. *pestis*, which includes the vast majority of virulent lineages that participated in the historically recorded pandemics (ANT, MED, ORI, and possibly, IN too); as well as six other clusters, including ssp. *caucasica*—0.PE2, ssp. *angolica*—0.PE3, ssp. *central asiatica*—0.PE4, spp. *tibetica*—0.PE7, ssp. *ulegeica*—0.PE5, and ssp. *qinghaica*—0.PE10.

## *Yersinia pestis* virulence determinants

Plague prevention requires a complete understanding of *Y. pestis* complex pathogenesis, which relies on a finely regulated arsenal of virulence determinants [36]. Among them, the best characterized ones are the *Yersinia* outer-membrane proteins (Yops), which constitute an array of effectors directly translocated into host cells through a type 3 secretion system (T3SS) [37–39]. The T3SS is encoded in the *Yersinia* virulence plasmid pYV/pCD1, which is also present in the enteric pathogens *Y. pseudotuberculosis* and *Y. enterocolitica*. As previously mentioned, the emergence of *Y. pestis* from *Y. pseudotuberculosis* is associated with the acquisition of the pFra and pPla plasmids, which encode critical virulence factors (see below), as well as major gene loss (gene deletions, SNP-based, or phase-variable/mutation-based pseudogenes) that had a major impact in adaptation to the flea vector [11]. Here, we will review virulence determinants for mammalian hosts.

**Fig. 2:**
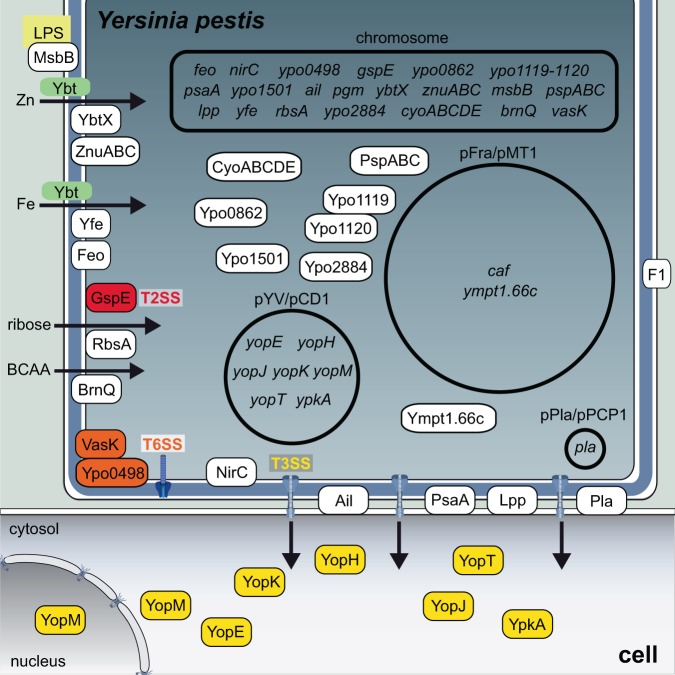
*Yersinia pestis* virulence determinants. *Y. pestis* requires the three well-characterized virulence plasmids pYV/pCD1, pPla/pPCP1, and pFra/pMT1, as well as chromosomally encoded virulence factors to cause disease. Examples of cytoplasmic, cell-surface associated, and secreted virulence factors are shown. The infectious process involves adhesion to host cells mediated by Braun lipoproteins Lpp and proteins such as Ail, PsaA, and Pla. Yop effectors, including YopH, YopE, YopT, YopJ, YpkA, YopM, and YopK, are subsequently delivered through the T3SS to trigger apoptosis, inhibit phagocytosis, and block cytokine production. LPS modification and the capsular antigen F1 encoded by the *caf* gene further contribute to *Yersinia* immune escape. The expression of many of these virulence determinants is induced during the transition from the temperature of the flea midgut (26 °C) to that of the mammalian host (37 °C). *Y. pestis* survival in the host requires efficient metal acquisition systems. The yersiniabactin-dependent iron uptake system is encoded in the high-pathogenicity island within the pigmentation chromosomal locus *pgm*. Other metal transport systems, including YbtX, ZnuABC, Yfe, and Feo, also play a role in infection. Additional virulence factors have been identified by signature-tagged mutagenesis, “per-pool” mutant screening, or in vivo transcriptional profiling, e.g., YMPY1.66c, BrnQ, RbsA, GspE, NirC, CyoABCDE, PspABC, Ypo0862, Ypo1119, Ypo1120, Ypo1501, and Ypo 2884. BCAA: branched-chain amino acids, Fe: iron, LPS: lipopolysaccharide, T2SS: type-two secretion system, T3SS: type-three secretion system, T6SS: type-six secretion system, Ybt: yersiniabactin, Yop: *Yersinia* outer-membrane protein, Zn: zinc. Components of the illustration are not drawn to scale

Yops have been shown to play pleiotropic roles during infection: they inhibit Rho GTPases and disrupt the actin cytoskeleton in order to inhibit phagocytosis, they downregulate the production of pro-inflammatory cytokines, and they induce cell death by multiple sophisticated mechanisms [40] (Fig. 2) (see also next section). For example, the tyrosine phosphatase YopH dephosphorylates focal adhesion complex proteins of immune cells [41]. YopE mimics GTPase-activating proteins (GAP) to downregulate host cell Rho GTPases [42]. The cysteine protease YopT cleaves and releases membrane-bound Rho GTPases [43]. YopJ is an acetyltransferase blocking both mitogen-activated protein kinase (MAPK) and nuclear factor-κB (NF-κB) signaling, inhibiting the production of pro-inflammatory cytokines [44, 45]. The serine/threonine kinase YpkA targets actin-regulating proteins and keeps Rho GTPases inactive through its guanine nucleotide dissociation inhibitor (GDI) domain [46]. YopM binds caspase-1 to arrest inflammasome assembly and activation [47], and behaves as a nucleomodulin modifying Interleukin-10 mRNA levels [48]. It is also an E3 ubiquitin ligase targeting NLRP3 and triggers host cell necrosis [49]. YopK is a particular virulence-associated factor that modulates the rate of injection of other Yops from within host cells [50] and also inhibits inflammasome activation [51]. It is important to highlight, as previously mentioned, that the function of several Yops has been studied only in *Y. pseudotuberculosis* or *Y. enterocolitica*, and remains to be investigated in *Y. pestis*.

Delivery of Yop effectors as well as cell invasion require adhesion of *Y. pestis* to host cells. Chromosomally encoded Ail outer-membrane protein contributes to cell attachment, Yop delivery, and serum resistance [52]. Another factor promoting Yop secretion is the Psa fimbriae (pH 6 antigen) that inhibit phagocytosis by macrophages and mediate *Yersinia* binding to epithelial cells by interacting with glycosphingolipids and phosphatidylcholine [53]. In contrast, the pFra/pMT1-encoded capsular antigen fraction 1 (F1) prevents bacterial uptake by inhibiting adhesion [54]. Subversion of host innate immunity also relies on abundant outer-membrane components, including the Braun lipoprotein Lpp [55] and the lipopolysaccharide, whose lipid A moiety is differentially acylated in a temperature-dependent manner by the acyltransferase MsbB (LpxM) [56]. The absence of the LpxL/HtrB acyltransferase in *Y. pestis* yields a tetra-acylated LPS, an antagonist for TLR4 receptors, which thus counteracts innate immunity [57]. The protease Pla, encoded in the 9.5-kb plasmid pPla/pPCP1, is another outer-membrane protein playing a key role in *Y. pestis* virulence through its adhesive, invasive, fibrinolytic, and coagulase properties. It has been shown to cleave multiple host cell substrates in vitro and/or in vivo, including plasminogen, alpha-2-antiplasmin, plasminogen activator inhibitor-1, urokinase plasminogen activator, C3 complement component, Fas ligand FasL, and the dual peroxidase/phospholipase peroxiredoxin [58, 59]. Pla, Ail, and F1 have been detected in outer-membrane vesicles, which may contribute to pathogenesis by dispersion and delivery of virulence factors [60].

Recently, novel virulence factors have been identified using a “per-pool” mutant screening method [61], as well as transposon sequencing (Tn-seq) and signature-tagged mutagenesis (STM) approaches [62, 63]. By investigating a library of *Y. pestis* mutants, the gene *ympt1.66c* (encoding a putative helicase) was identified as required for bacterial intracellular survival during the early stages of bubonic plague [61]. Besides known virulence genes, genome-wide fitness profiling led to the identification of more than 30 genes involved in nutrient acquisition and metabolism, which were necessary for infection, including the branched-chain amino-acid importer BrnQ [62]. Using STM and a murine model of pneumonic plague, a total of 118 mutants were found to have a lower capacity to colonize the spleen [63]. A secondary screen revealed that 20 out of the 118 individual mutants displayed attenuated virulence similarly in a murine model of bubonic plague: mutations were present not only in Pla but also in proteins of unknown function that require further characterization. Moreover, the study uncovered roles in *Y. pestis* pathogenesis for the type 6 secretion system (T6SS) component VasK and the ribose transport system ATP-binding protein RbsA [63]. Additional T6SS components have been shown to play an important role in macrophage cytotoxicity, resistance to phagocytosis, and in a murine model of pneumonic plague [64]. In the same study, four new factors identified by STM were confirmed to contribute to virulence in mice, namely the general secretion pathway protein E of the type 2 secretion system GspE, the βγ crystallin superfamily protein Ypo2884, the cytochrome *o* oxidase CyoABCDE, and the multifunctional Tol-Pal system proteins Ypo1119–1120 [64]. Further investigation will be required to understand their role in pathogenesis.

A critical property of bacterial pathogens is their capacity to overcome nutritional immunity, i.e., host sequestration of essential metals such as iron and zinc [65], a mechanism which can protect against plague [66]. To acquire iron, *Y. pestis* secretes the siderophore yersiniabactin (Ybt), which has been shown to be essential for bubonic and pneumonic plague [67]. In addition, the Yfe and Feo metal transporters play a role in bubonic plague [68]. Interestingly, the Ybt biosynthetic machinery is also necessary for zinc uptake by *Y. pestis*. Together with Ybt, the ABC transporter ZnuABC and YbtX, a member of the major facilitator superfamily, contribute to infection in murine models of plague [69–71]. YbtX was among the five new virulence factors involved in the development of pneumonic plague in mice that were identified using in vivo transcriptional profiling of *Y. pestis* [70]. The role of the other four factors, the phage-shock system PspABC, the protein of unknown function YPO0862, the putative esterase YPO1501, and the nitrite transporter NirC, remains to be defined.

## *Yersinia pestis* and subversion of host innate immunity

One of the most surprising features of *Y. pestis* infection, either through a flea bite or through contamination via respiratory droplets, is the brutal transition from an absence of immune response and clinical symptoms, to a bursting inflammation and fatal sepsis with abundant bacteria in the body. This lag period has been termed “pre-inflammatory phase” and benefits *Y. pestis*, which migrates toward lymph nodes (bubonic plague) or lungs (pneumonic plague) to multiply silently. This immune evasion is exerted by several mechanisms that include the T3SS-mediated neutralization of immune cells (see the previous section), the absence of detectable pathogen-associated molecular patterns (PAMPs [57]), and modulation of host innate immune cell interactions (detailed below).

Neutrophils are a very early line of innate defense and inflammation at infection sites. It was commonly accepted that *Y. pestis* is eliminated effectively when phagocytosed by neutrophils, but survives and replicates when phagocytosed by macrophages. It has now been shown that *Y. pestis* grown at flea temperature (close to that of the host skin) at which the T3SS is not yet upregulated, can survive and replicate within human neutrophils [72]. *Y. pestis* can also transit from this first niche to a second one when macrophages phagocytose infected neutrophils in a cleaning process named efferocytosis [73]. Within macrophages, *Y. pestis* can survive in autophagosomes [74], and it has recently been shown that *Y. pestis* is also capable of inhibiting phagosomal maturation by perturbing the host endosomal recycling pathway, allowing bacterial intracellular replication [75, 76]. Efferocytosis is accompanied by secretion of IL-1RA [73], a cytokine exerting anti-inflammatory effects by blocking IL-1R1 signaling: this strategy would allow *Y. pestis* to reduce innate immunity mechanisms and multiply, meanwhile upregulating its T3SS. Then, in target organs, *Y. pestis* would preferentially inject Yops in neutrophils by specifically recognizing their surface complement receptor 3 [77] and resists neutrophils toxic effectors through the activity of the lysozyme inhibitor Ivy [78] and the two-component regulatory system OmpR-EnvZ [79].

How *Y. pestis* disseminates from skin flea injection sites to lymph nodes was unknown and it was suspected that phagocytes (i.e., macrophages, dendritic cells, or neutrophils) could carry the bacteria. This scenario has been challenged by recent observations in murine models of infection, suggesting that the *Y. pestis* spread from the skin is very rapidly achieved [80] in the absence of interactions with host cells [81], suggesting therefore that bacterial transport to the draining lymph nodes can be driven solely by lymph flow [82]. However, an intracellular carriage by dendritic cells and monocytes motivated principally by sphingosine-1 phosphate (S1P)-driven chemotaxis toward lymph nodes was also reported, that probably occurs more slowly [83].

Within the infected lymph nodes, several pathways of cell death have been proposed to be activated by *Y. pestis* [84]. Applying murine *Y. pseudotuberculosis* infection models, it has been suggested that the T3SS effector YopJ induces macrophage apoptosis [85], a noninflammatory mechanism required for bacterial systemic infection [86]. However, in a bubonic rat infection model, YopJ-induced apoptosis was not required for *Y. pestis* virulence [87]. Necroptosis has been proposed to be required for *Y. pestis* dissemination from lymph nodes during the pre-inflammatory phase: cell membrane rupture leads to bacterial release and subsequent capture by phagocytes chemotactically attracted by S1P [88]. Bacterial intracellular carriage by dendritic cells and monocytes would then mediate *Y. pestis* node-to-node trafficking [83]. Of note, it has been reported that *Y. pseudotuberculosis* redirects apoptosis to caspase-1-dependent inflammatory pyroptosis in pre-activated cells [89], and *Y. pestis*-induced pyroptosis results from YopJ blockade of kinase TAK1, which activates caspase-8 and pore-forming gasdermin D [90]. Paradoxically, other Yops (YopM and YopK) oppose to pyroptosis by preventing inflammasome and caspase-1 activation [47, 51]. How *Y. pestis* could induce necroptosis also requires clarification, as caspase-8 cleavage, the signal for *Y. pestis-*induced cell death [91], has been reported to inhibit necroptosis [92]. Interestingly, caspase-8 also controls antibacterial immunity and Toll-like receptor-induced cytokine production, and mice lacking caspase-8 are highly susceptible to *Yersinia* infection [93].

Despite being caused by the same pathogen, pneumonic plague differs from bubonic plague by the mode of infection (aerosols versus fleas), the target tissue (lungs versus skin/lymph nodes), and ultimately pathophysiology and lethality. The pre-inflammatory phase during pneumonic plague generates a permissive environment that allows *Y. pestis* growth [94]. Resident alveolar macrophages are targeted early in infection, followed by preferential targeting of neutrophils in the later stages [95]. Inflammasome-dependent IL-1β/IL-18 release occurs early after bacteria enter the lung, but fails to cause inflammation due to the simultaneous release of anti-inflammatory IL-RA blocking IL-1 receptors [96]. Therefore, and interestingly, IL-1RA thus seems to play a key role in establishing the pre-inflammatory phase of both bubonic and pneumonic forms of plague [73, 96]. In the latter, during the pro-inflammatory stage associated with clinical symptoms, neutrophils attracted to lungs are responsible for the pneumonia and necrosis destroying the lungs [95]. In this context, the T3SS effector YopK is a critical modulator of macrophage apoptosis and promotes progression of pneumonic plague toward the inflammatory stage [97]. An important distinction between bubonic and pneumonic plague is the temperature of the environment in which the bacteria grew prior to transmission: *Y. pestis* infecting lungs via aerosols that initially grew at 37 °C in the contaminating patient’s lungs [98], have therefore an upregulated T3SS and are thus more able than bacteria grown below 30 °C in fleas to kill host cells. It has been shown that YopM inhibits the death of neutrophils in the center of lung lesions, preventing the release of their bactericidal and inflammatory content [99]. In consequence, as in the bubo, a delay in cell death contributes to the lag before the pro-inflammatory phase.

The existence of a pre-inflammatory phase during plague suggested that intervention to trigger a faster response of innate immunity could possibly counteract infection, as first indicated by an earlier report that inflammation resulting from a strong lipopolysaccharide response overcame *Y. pestis* virulence [57]. Two recent studies further show that specifically fostering the action of neutrophils or macrophages, respectively, at the very onset of the disease can control *Y. pestis* infection process. In mouse lungs, a cytokine-induced recruitment of neutrophils reduced bacterial counts and improved survival from pneumonic plague [100]. In a mouse bubonic plague model, transferring M1-polarized macrophages also enhanced survival [101].

**Fig. 3:**
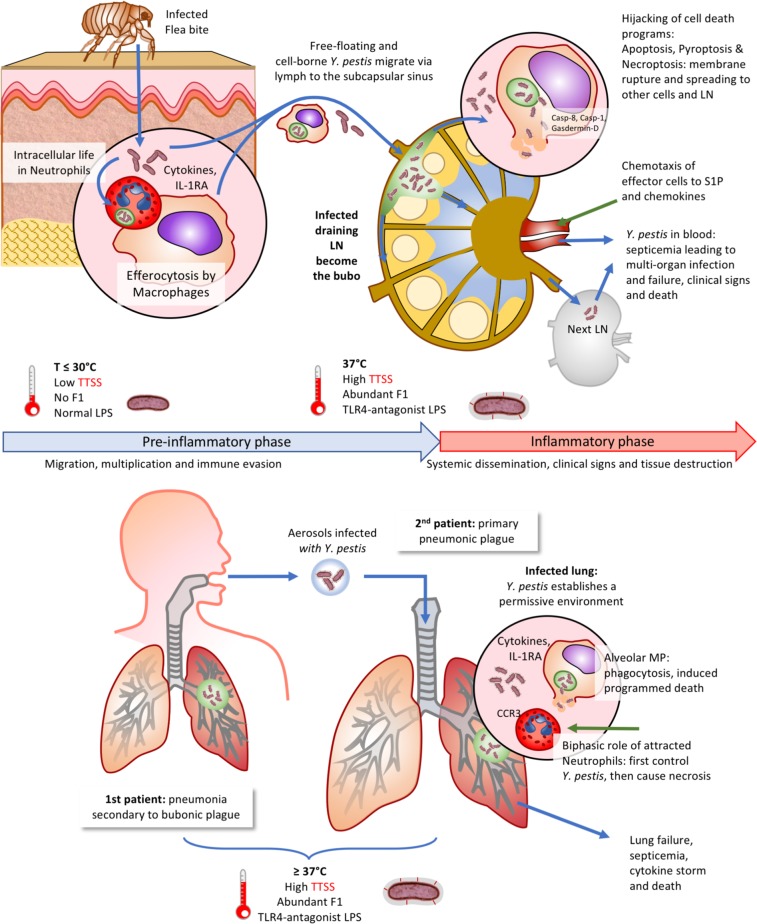
Innate immunity subversion by *Yersinia pestis*. Schematic representation of *Y. pestis* transmission and migration in the human body during bubonic (upper panel) and pneumonic plague (lower panel). The effect of temperature of origin and its effects on the bacterium are indicated at various steps. A focus is given on innate immunity host cells encountered by the pathogen and involved in immune evasion mechanisms described in the text, which are shown as zoomed areas

Innate immunity mechanisms are amplified when adaptive immunity is effective, such as after vaccination (see next section), and contribute to protection. In that regard, several recent observations point to a role for IL-17 in both wings of this response. During the innate response, IL-17 acts by inducing production of antimicrobial peptides and attracting neutrophils [102], as well as by activating macrophages to produce IFN*γ* [103]. IL-17-producing T lymphocytes (Th17 cells) have been reported to be essential to cure pneumonic plague [104]. Surprisingly, a mechanism of Th17 induction may be injection of Yops into naive T cells, as observed with *Y. pseudotuberculosis* [105]. In agreement, vaccination using a live attenuated *Y. pestis* NIIEG strain induces a Th17 polarization in humans [106]. Moreover, Th17 cells may develop in parallel to Th1 ones, as was observed with a live *Y. pseudotuberculosis* oral plague vaccine [107]. Therefore, IL-17 appears important against plague, and how it is limited during the pre-inflammatory phase needs to be determined (Fig. 3).

## Vaccination against plague

Despite considerable progress in prevention and cure, natural foci still exist in Africa, Asia, and America, and plague is endemic in more than 25 countries worldwide, which would benefit from vaccination. No new vaccine has recently been licensed in Europe or in the United States to replace the killed whole-cell *Y. pestis* formerly produced in the United States and Australia, which was discontinued because of its short-lasting protection and low efficiency. The live *Y. pestis* EV vaccine previously used with benefit in Madagascar, and still used in Asia and Russia, was never licensed in occidental countries. Most current efforts are focused on vaccines against pneumonic plague, considering *Y. pestis* as a potential biowarfare agent. The most advanced candidates are molecular vaccines developed by the United States, the United Kingdom, and China governmental agencies, and are patented but not yet licensed. They combine *Y. pestis* F1 and V antigens and are called rF1-V, RypVax, and SV1, respectively. F1 is the main protein component of the pseudocapsule, and is a dispensable virulence factor. The V antigen (LcrV) is an essential part of the T3SS. Two doses of these F1/V vaccines provide 100% protection to mice against bubonic and pneumonic plague [108–111]. However, when F1-V was tested in nonhuman primates, *Cynomolgus* macaques could be protected against aerosolized *Y. pestis*, but not African green monkeys [112]. Therefore, it is not yet clear whether F1-V would reliably provide protection to humans. Because rF1-V and SV1 have completed phase I and II trials, WHO recently gathered experts to define principles on how to build phase III plague vaccine efficacy trials in endemic countries [113]. rF1-V is announced for 2021 for use by the US army and licensure in the United States can be obtained following FDA’s “Animal rule”, a procedure which applies when traditional efficacy trials in humans are unethical or impractical [114].

Several other vaccine candidates have been proposed. Many *Y. pestis* strains attenuated by genetic engineering are immunogenic and confer protection, but none was brought to the clinical evaluation phases. Another molecular vaccine, patented by the University of Chicago and called V10, is composed of a shorter V antigen only, and provided 100% protection against bubonic and pneumonic plague to mice and *Cynomolgus* macaques [115]. Live vector vaccines producing F1 and V have also been developed, using the modified Vaccinia virus (strain Ankara: MVA) or attenuated *Salmonella* as a vector. However, these vaccines did not progress to clinical trials. A live *Y. pseudotuberculosis* may also be a valuable vaccine against plague [116, 117]. We developed a live attenuated *Y. pseudotuberculosis* strain producing the F1 pseudocapsule, named VTnF1 [107]. Because a single oral dose provided protection of mice against bubonic and pneumonic forms caused by high doses of *Y. pestis*, VTnF1 appears as a very promising plague vaccine candidate.

## Plague diagnostics: lessons from the 2017 pneumonic plague outbreak in Madagascar

As previously mentioned, pneumonic plague is transmitted from person to person through respiratory droplets. After an incubation period of 24–96 h, the disease progresses rapidly and is nearly always fatal in a few days in the absence of an early antimicrobial treatment. From August to November 2017, Madagascar experienced an unprecedentedly large pneumonic plague outbreak with multiple foci, including two main urban areas: Antananarivo, the capital, and Toamasina, the main seaport [10]. A total of 2414 suspected cases were reported. Spread of pneumonic plague was of great concern as its propagation is favored by the closeness of people, especially in the densely populated cities. Health authorities urgently had to organize the screening of the suspected patients, the collection of samples for biological diagnosis, the prompt treatment of patients, and the follow-up of the contacts. In the context of this outbreak, light was brought toward the specific problems associated with the diagnosis of pneumonic plague.

Screening of the suspected patients was based on epidemiological and clinical criteria, as described in the case definition provided by the World Health Organization (WHO) [118] and biological samples were collected at the points of care. Biological diagnosis of pneumonic plague highly relies on the quality of the sample: since pneumonic plague is a lower respiratory tract infection, deep respiratory secretions are required for biological tests, not saliva or spit. However, during an outbreak, the aim is to diagnose the disease during the invasion phase before the onset of severe symptoms. Thus, producing good-quality sputum is often difficult for patients with mild symptoms and also for children. Moreover, since sputum specimens can be viscous and thick, liquefaction and homogenization are fully required before execution of some specific biological assays [119] (see below). However, because of lack of reagents, equipment, and trained staff, this first step of the process is seldom performed at local points of care, resulting in wrong interpretations of subsequent biological assays. The rapid diagnostic test (RDT), based on the detection of the F1 antigen of *Y. pestis* [120], is a practical method that can be implemented by trained staff at local points of care, can provide results within 15 min, and has been validated for diagnosis of bubonic plague. Nevertheless, its performances in sputum still need to be evaluated. Indeed, false-negative and false-positive results may be observed with sticky expectorations, due to absence or incomplete sample migration along the dipstick. False-negative results may also occur with saliva. Therefore, clinically pneumonic plague-suspected patients must be treated without considering the result of the bedside RDT.

**Fig. 4:**
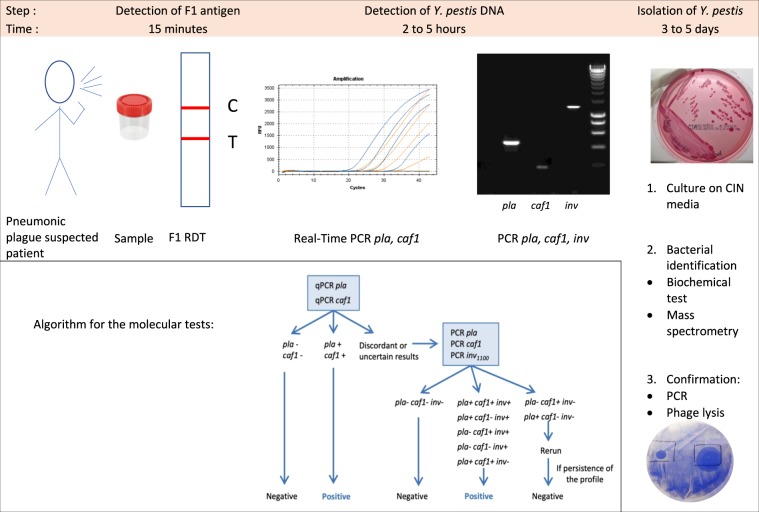
Plague diagnostics: from presumption to confirmation. Upon plague suspicion, according to epidemiological and clinical criteria, collected samples (sputum, expectoration) can be analyzed for the detection of the *Y. pestis* capsular antigen F1 using the rapid diagnostic test (RDT: the C band is a migration control, and the T band reveals the presence of F1). Confirmation is performed through detection of *Y. pestis* DNA using real-time PCR targeting *pla* and *caf1*, and in the case of discordant or uncertain results, a PCR targeting *pla, caf1*, and *inv* is performed. Isolation of *Y. pestis* remains the gold standard in biological diagnostic tests: culture on selective CIN media is followed by bacterial identification using biochemical tests or mass spectrometry, confirmation using PCR, and phage lysis. Boxed area: algorithm for molecular tests using qPCR and PCR (reprinted from ref. [135])

Molecular biology tests have been developed in order to reduce the delay and increase the sensitivity of diagnostics (Fig. 4). Conventional polymerase chain reactions (PCR) targeting the *pla*, *caf1*, *inv*, and *yopM* genes [121, 122] reduce the delay of diagnostics to 3–4 h, while real-time PCR can be performed in only 2 h. The *pla* gene is located on the pPla/pPCP1 plasmid, which is present in 150–200 copies per bacterium [123], resulting in a high sensitivity. A real-time PCR targeting *pla* in the sputum [124] displays a sensitivity of 100 cfu/ml in spiked sputum. However, *pla* may also be found in other Enterobacteriaceae such as *Citrobacter koseri* and *Escherichia coli* [125] and these bacteria may be present in the sputum. Therefore, the sputum must be tested by multiplex real-time PCR, targeting additional genes. The *caf1* gene is located on the pFra/pMT1 plasmid, which is considered specific to *Y. pestis*, but it is present in only about two copies per bacterium [123]. A multiplex real-time PCR on *pla* and *caf1* has been described [126] but it was tested only in patients with suspected bubonic plague (not on sputum). The *yopM* gene, located on the *Yersinia* virulence plasmid pYV/pCD1, is present in about four copies per bacterium, but pYV may also be found in *Y. pseudotuberculosis* and pathogenic *Y. enterocolitica* strains. The *inv* gene is a chromosomal gene present in *Y. pestis* and *Y. pseudotuberculosis*; an insertion in *Y. pestis* made it larger [127] and a conventional PCR may distinguish it by the amplicon sizes produced in *Y. pseudotuberculosis* (400 bp) compared with *Y. pestis* (1100 bp). During the 2017 pneumonic plague outbreak in Madagascar, the strategy chosen to detect *Y. pestis* DNA was first to test the samples by a multiplex real-time PCR targeting *pla* and *caf1*, and to confirm the uncertain cases by a conventional PCR targeting *pla*, *caf1*, *inv*_*1100bp*_, and *yopM*. It is worth mentioning that the chromosomal gene *ypo2088* is specific to *Y. pestis* and spiked expectorations have been successfully tested by real-time PCR targeting this gene [128]. Molecular biology tests are usually performed in hospitals and research institutions; however, portable real-time PCR instruments are being developed and could be helpful in remote plague endemic areas [129]. Assays relying on the loop-mediated isothermal amplification (LAMP) technology have also been developed [130], but they still remain to be evaluated on sputum.

Among the biological diagnostic tests, microbial isolation of *Y. pestis* remains the gold standard. Although *Y. pestis* can grow on usual culture media, the use of a selective agar supplemented in cefsulodin–irgasan–novobiocin (CIN) favors the isolation of the bacterium in polymicrobial samples such as sputum. WHO recommendations are to rinse the mouth out with water prior to sample collection in order to reduce the contaminations by the oral flora [131]. After 2- or 3-day incubation at 28 °C, suspected colonies on CIN agar may be identified by biochemical tests, PCR, and *Y. pestis*-specific phage lysis. Automated identification systems are more and more often used in the laboratories: they are fast and can efficiently identify a large variety of bacteria; however, misidentifications of *Y. pestis* have been reported [132]; therefore, a reference laboratory must always confirm the taxonomic assignation.

## Concluding remarks

Due to its exceptional virulence, its multiple modes of transmission and pathogenesis, its outstanding skills to escape host molecular and cellular immunity mechanisms, and the indelible mark it has left on human societies in the past, *Y. pestis* is a remarkable model to study infection and the evolution of bacterial pathogenicity. The recent improvement in high-end technologies, allowing whole-genome sequencing and more resolution in vitro as well as in vivo microscopy, has directly benefitted the understanding of various aspects of plague, including bacterial phylogeny, pandemics history, pathogenesis mechanisms, and subversion of immune responses.

However, several aspects of plague natural history are still incompletely understood. The ecology of *Y. pestis* in the environment remains to be fully explored, as novel potential reservoirs are discovered [133, 134]. Some molecular mechanisms of virulence of *Y. pestis* have been inferred from comparisons with other pathogenic *Yersiniae*, including the enteric pathogens *Y. pseudotuberculosis* and *Y. enterocolitica*, which might bias our understanding of plague. More studies using fully virulent *Y. pestis* strains are required to completely grasp the physiopathology of the disease. The bacillus discovered 125 years ago by Alexandre Yersin has still a lot of secrets to reveal.
